# In vivo and in vitro expression of five genes involved in *Corynebacterium pseudotuberculosis* virulence

**DOI:** 10.1186/s13568-018-0598-z

**Published:** 2018-05-30

**Authors:** Jefferson Ivan Corrêa, Andreas Stocker, Soraya Castro Trindade, Vera Vale, Thais Brito, Bruno Bastos, José Tadeu Raynal, Patrícia Mares de Miranda, Adriano Costa de Alcantara, Songeli Menezes Freire, Lília Moura Costa, Roberto Meyer

**Affiliations:** 1 Department of Biointeração-Health Sciences Institute, Federal University of Bahia, Salvador, Brazil; 2 Laboratório de Imunologia e Biologia Molecular, Instituto de Ciências da Saúde, Universidade Federal da Bahia, Avenida Reitor, Miguel Calmon, S/N, Vale do Canela, Salvador, Bahia CEP 40.110-100 Brazil; 3 Laboratory of Infectology-Prof., Edgard Santos University Hospital, Federal University of Bahia, Salvador, Brazil

**Keywords:** *Corynebacterium pseudotuberculosis*, Proteins, Genes, Virulence factor

## Abstract

Caseous lymphadenitis (LC) is a chronic contagious disease caused by *Corynebacterium pseudotuberculosis*, which mainly affects goats and sheep. Vaccination is an effective but not yet well-established method, partly due to a lack of knowledge surrounding the most effective immunoprotective components. The present study aimed to quantify and compare the in vivo expression of genes *pld* (phospholipase D), *cpp* (CP40), *nanH* (neuraminidase H), *sodC* (superoxide dismutase C) and *spaC* (adhesin) using qRT-PCR, with the respective expression in vitro. Caseous material of abscesses removed from five animals was cultured, with colonies suggestive of *C. pseudotuberculosis* identified. RNA extraction was performed on these samples, as well as on the respective pellets derived from liquid cultures brain heart infusion. After evaluating RNA integrity, complementary DNA was synthesized, followed by the relative quantification each of the genes of interest. Mean mRNA expression of the five genes found in abscesses and in cultures differed significantly, with respective values of: *nanH* 811.50 ± 198.27 and 359.35 ± 75.45 (p = 0.009); *cpp* 856.31 ± 385.11 and 154.54 ± 94.34 (p = 0.0039); *plD* 922.70 ± 450.73 and 212.41 ± 153.10 (p = 0.016); *sodC* 1,293.53 ± 564.75 and 223.63 ± 145.58 (p = 0.016); *spaC* 1,157.10 ± 525.13 and 214.26 ± 125.70 (p = 0,016). Expression was observed to be 6–8 times higher in abscesses than in cultures, Indicative that is a genetic expression of the in vitro bacterium exists, yet in vivo has a greater magnitude corroborating to one of these virulence factors in the pathogenesis of LC.

**Electronic supplementary material:**

The online version of this article (10.1186/s13568-018-0598-z) contains supplementary material, which is available to authorized users.

## Introduction

Caseous lymphadenitis (CL), a chronic contagious disease that affects small ruminants, is caused by *Corynebacterium pseudotuberculosis*, a gram-positive, pleomorphic, non-sporulated, facultatively anaerobic bacillus belonging to the C*orynebacteriaceae* family (*Actinomycetales* order).

The literature is inconsistent with regard to the prevalence of this disease in goat and sheep herds. In Brazilian goats raised in semi-arid areas, studies conducted by Unanian et al. (1985) reported a prevalence of 12% when sampling animals destined for slaughter (internal abscesses) and 42% when considering animals used extensively for breeding (superficial abscesses). In Bahia, a prevalence of 55.3% was observed in sheep of a commercial herd with a flock of 2500 animals (Bastos et al. 2011), as a serum-prevalence of 22.1% in sheep breeders sampled in agricultural expositions (Nascimento 2012). Another study conducted by Brown et al. (1987) in Brazil found an incidence of 14% in slaughter animals. In Australia, Batey (1986) found an 8% incidence of this disease in slaughterhouses. Infection rates ranged from 6.31 to 52% in sheep inspected at slaughterhouses in Australia, Canada, the United States and Brazil (Batey 1986; Middleton et al. 1991; Arsenault et al. 2003; Stoops et al. 1984; Sá et al. 2013; Souza et al. 2011). Meyer (2003), in an extensive serological investigation involving 1966 goats conducted in 19 municipalities throughout the semi-arid zone of the state of Bahia, found highly variable prevalence, with infection rates ranging from 9.2 to 72.2%. Considering that most goat herds are concentrated in the northeastern region of Brazil, together with the fact that these animals are essential for the sustenance of small producers, caseous lymphadenitis poses a relevant threat to the raising of livestock in this region (Meyer 2003).

The strategy to control caseous lymphadenitis is mainly based on vaccination, which has yet to be widely applied throughout the region, in addition to the adoption of complementary measures concerning the inclusion of new animals in herds, early detection of infected animals, segregation, treatment or elimination of diseased animals, special care in routine handling, as well as other measures (Guimarães et al. 2011; Windsor 2011; Kumar et al. 2013).

The immunogenic components of the most effective commercially available vaccines are attenuated live cells of *C. pseudotuberculosis*, which may be mixed with innocuous forms (toxoids) of phospholipase D, the main virulence factor of *C. pseudotuberculosis* (Meyer 2003).

Additional virulence factors were proposed following the sequencing of the *C. pseudotuberculosis* FRC41 genome by Trost et al. (2010). Analysis of the chromosomes in this strain allowed for the identification of several genes highly likely to be involved in virulence, including *cpp* (CP40), *nanH* (neuraminidase H), *rpfA* and *rpfB* (resuscitation-promoting factors A and B), *nor* (nitric oxide reductase), *dtsR2* (acetyl-CoA carboxylase, beta subunit, involved in the biosynthesis of mycolic acid) and *spaC* (the SpaC protein, a component of adhesin). Although *sodC* gene was not included in the list of potential virulent factors in the strain sequenced by Trost et al. (2010, p. 10), several researchers consider superoxide dismutase (SodC) to be a virulence factor (Sanjay et al. 2010; Suo et al. 2012). The present study aimed to detect and compare, using quantitative real-time PCR with reverse transcription (qRT-PCR), the in vivo expression of genes *pld*, *cpp*, *nanH* and *sodC* with expression in vitro. Since bacterial adhesins are virulence factors (Petri et al. 2010) the *spaC* gene, which synthesizes a protein located at the tip of *C. pseudotuberculosis* adherent pilli, was also included in the study.

## Materials and methods

### Abscess sampling

Samples were collected from five animals on a farm in the municipality of Jaguarari, Bahia-Brazil, which were diagnosed with caseous lymphadenitis. After removing the wool, the skin covering the lesions suspected of CL was disinfected with 5% iodized alcohol. Next, a sterilized scalpel was used to make an incision in the lesion, and the caseous content was then collected in a wide-mouthed sterile flask. Aliquots of approximately 200 µL were quickly transferred to cryotubes, which were immediately immersed in dry ice. The remaining caseous material was kept at room temperature. All samples obtained from the animals were transported to the city of Salvador on the same day of collection. The five samples kept on dry ice were rapidly transferred to a freezer and stored at − 80 °C, while the five samples kept at room temperature were sent to the Bacteriology Laboratory at UFBA (the Federal University of Bahia) for culturing and subsequent identification.

### Bacteriological analysis

The five samples submitted to culturing were seeded on sheep blood agar plates and incubated at 37 °C for 24–48 h. Colonies suggestive of *Corynebacterium pseudotuberculosis* were identified by colonial morphology, hemolysis pattern analysis, Gram staining and the use of the following biochemical tests: glycose, sucrose, lactose and maltose fermentation, as well as urease and catalase production. Following the identification of *C. pseudotuberculosis* isolates, samples were cultured placed in BHI broth containing 0.1% Tween and incubated at 37 °C for 48 h. During the exponential growth phase, liquid culture (500 µL) was transferred to 1.5 mL microtubes in triplicate and centrifuged at 4 °C for 10 min. The supernatants were discarded, while the pellets were stored at − 80 °C until the time of total RNA extraction.

### RNA extractioRNA extraction was performed using a commercially available kit

RNeasy Mini Kit (Qiagen, Austin, Texas, USA). Cryogenic vials containing approximately 200 µL of caseous content or culture pellets previously stored at − 80 °C were allowed to thaw spontaneously at room temperature. Immediately after, thelysis buffer included in the kit was added to the pellets and, after homogenization, the entire liquid content was transferred to new cryogenic vials containing eight beads of stainless steel. Around 40–50 µg of caseous content were placed in identical cryogenic vials (containing the same amount of beads), followed by the addition of an identical quantity of lysis buffer. The tubes were vertically agitated for 5 min in a Speedmill homogenizer (Analytic Jena AG) to rupture the bacterial cells. The tubes were then centrifuged at 16,000*g* for 3 min and the supernatants were transferred to silica columns for RNA recovery. All samples containing abscess material, as well as the respective pellets derived from liquid cultures, were extracted in the same experiment in accordance with the kit manufacturer’s instructions. Upon termination of the extraction process, 40 µL of eluate from each sample were preserved at − 80 °C until the time of reverse transcription. The DNA extraction of samples containing macrophages infected with *C. pseudotuberculosis* was performed at the Professor Edgar Santos University Hospital (HUPES) in Salvador-Bahia. These samples were transported in dry ice and immediately transferred to a freezer and kept at − 80 °C until the time of complementary DNA production.

### cDNA production

cDNA production was performed using 3 µg/µL of random hexamers (Invitrogen, Carlsbad, CA, USA) and 200 U/µL of Superscript III Reverse Transcriptase enzyme (Invitrogen, Carlsbad, CA, USA). Briefly, 3 µL of eluate obtained from sample extract were added to 5.7 µL of ultrapure water with 4 µL of dNTPs and 0.80 µL of random hexamers. Tubes containing a final volume of 13.5 µL of this mixture were placed in a thermal cycler at 95 °C for 5 min to separate the RNA strands present in the solution. Next, a second mixture was made containing 4 µL of First Strand Synthesis Buffer, 1 µL of dithiothreitol, 1 µL of RNAse inhibitor and 0.5 µL of Superscript III RT. After the tubes containing the first mixture were removed from the thermocycler and kept in a freezer at − 4 °C for 3 min, 6.5 µL of the second mixture was added to each tube. After annealing was completed, extension was performed as follows: 10 min at 25 °C, 30 min at 50 °C, 15 min at 53 °C and 10 min at 55 °C. Next, reverse transcriptase inactivation was carried out by incubating the tubes at 70 °C for 15 min. The cDNA product was maintained at − 80 °C until the time of qPCR to quantify the genes investigated herein.

### RNA integrity verification

Three liquid cultures of *C. pseudotuberculosis* in exponential growth phase were incubated in BHI with 0.1% Tween. Following RNA extraction, the three eluates of the samples with higher expression were subsequently submitted to the cDNA protocol described above. To assess RNA integrity, each sample of this material underwent conventional PCR (Taq polymerase, Invitrogen, Foster City, CA, USA) at a final volume of 25 µL, containing: 15.15 µL of ultrapure water, 2.5 µL of magnesium-free buffer (10×) 2.0 µL of dNTPs (4 × 2.5 mM), 1.25 µL of MgCl_2_ 50 mM, 0.75 µL of each of the following primers: Euba-01-F and Uni-R-1492 10 µM, 0.1 µL of Platinum Taq Polymerase 5 U/µL and 2.5 µL of cDNA. These mixtures were incubated for 2 min at 94 °C in a thermocycler (Eppendorf), and then submitted to 45 staggered cycles as follows: 94 °C for 20 s, 61 °C for 60 s and 72 °C for 60 s, followed by a 4-minute extension at 72 °C.

Electrophoresis was subsequently performed in 1.0% agarose gel pre-stained with SYBR Safe (Invitrogen, Paisley, UK). In the same gel, a DNA ladder (Invitrogen, Carlsbad, CA, USA) containing 100–2000 base pairs was added. Photographic documentation was employed to verify the presence of a 1492 bp band.

### qPCR

Paired mRNA quantification with respect to each gene studied was performed by loading a thermocycler with optical tubes of either abscess samples or cultures of abscesses obtained from the same animals. Accordingly, 5 abscess samples and 5 culture samples were analyzed to compare the expression of the five genes encoding virulence factors and the 16S RNA housekeeping gene. All reactions were performed at a final volume of 25 µL containing: 7.4 µL of RNase-free water, 12.5 µL of Power SYBR Green Master Mix (2X) (Applied Biosystems), 1 µL of Bovine Serum Albumin (1 mg/mL), 0.75 µL of 10 µM forward primers and 0.75 µL of 10 µM of reverse primers, 0.1 µL of Platinum Taq Polymerase enzyme (5 U/µL) and 2.5 µL of cDNA eluates.

**Fig. 1:**
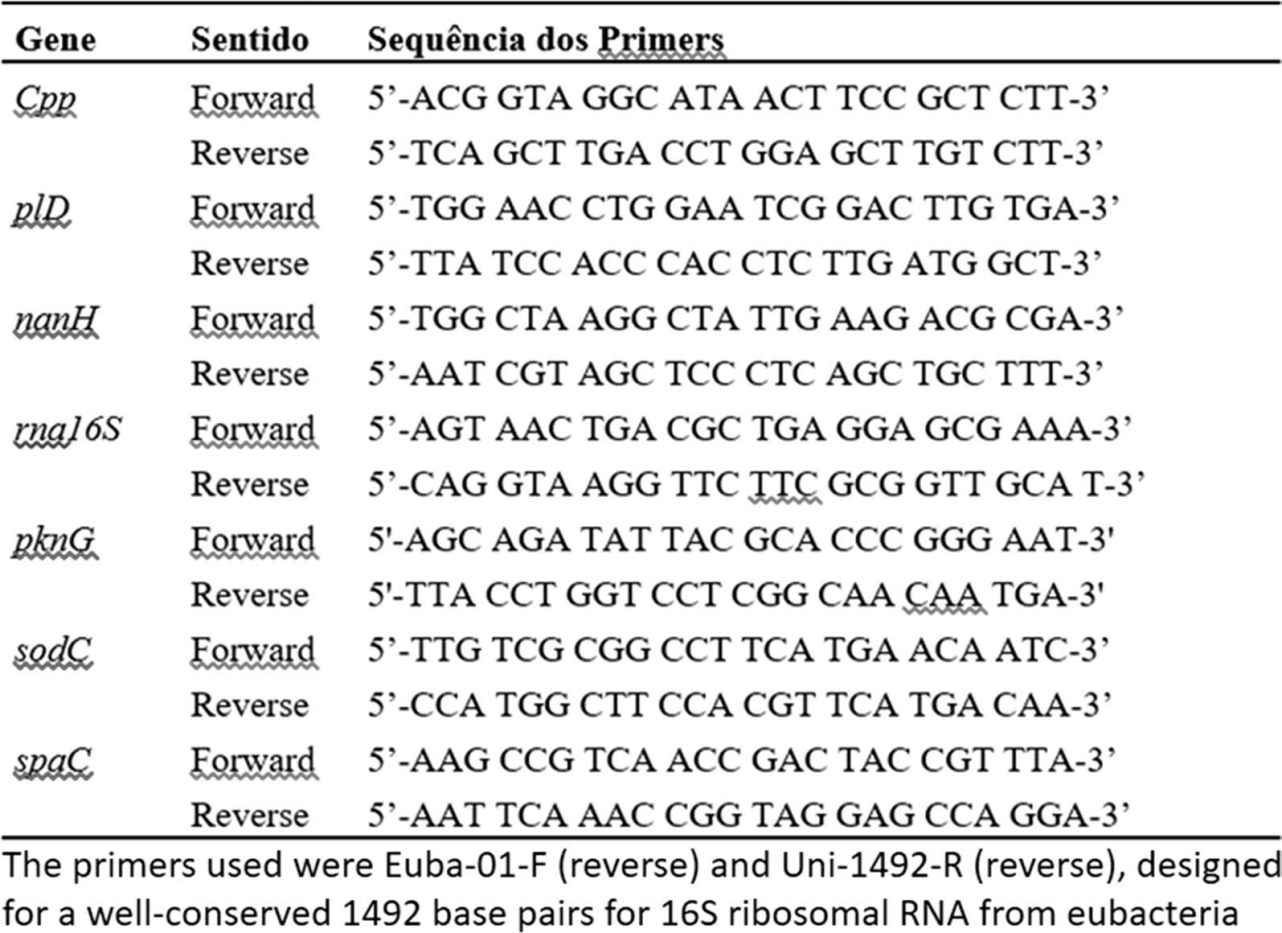
Primers used in the experiments

The primers utilized are shown in Fig. 1 Primer pairs were designed using the Primer Quest tool found on the Integrated DNA Technologies-ITD website, (http://www.idtdna.com/site). Seven pairs of primers were chosen for sequences ranging in size between 250 and 300 base pairs in order to standardize the dissociation temperatures to approximately 63 °C, allowing all qPCR reactions to be performed in a single round (Fig. 1).

Target amplification was performed using an ABI Prism 7500 thermocycler (Applied Biosystems) under the following time and temperature conditions: initial denaturation for 10 min at 95 °C, followed by 45 cycles of denaturation for 20 s at 94 °C and annealing for 60 s at 62 °C. The result of each qPCR amplification was evaluated by analyzing the dissociation curve (melting curve).

### mRNA quantification

Virulent Corynebacterium pseudotuberculosis in exponential growth phase was cultured (BHI broth with 0.1% Tween), then homogenized and centrifuged at 6000*g* for 5 min at 4 °C, after which the supernatant was discarded. The pellet was preserved in a freezer at − 80 °C and nucleic acids were extracted using a commercially available RNeasy Mini Kit (Qiagen). The eluate (40 µL) was aliquoted and used for cDNA production, in accordance with procedures described above. Six serial dilutions were prepared with ultrapure water for PCR, with cDNA ranging from 10^−1^ to 10^−6^. The original solution and all six dilutions were frozen at − 80 °C until the quantitative reactions were performed. For each of these dilutions, six amplifications were performed using specific primers for each of the five genes studied (see Table 1) plus another pair of primers for the 16S ribosomal RNA gene, used as a control for being one of the genes most used in studies of bacterial gene expression (Carvalho et al. 2014). After qPCR, linear regression analysis was applied to generate a calibration curve for each of the genes.

**Table 1:** Relative quantification and exression multiplicity of *nanH*, *cpp*, *pld*, *sodC* and *spaC* genes from *Corynebacterium pseudotuberculosis*

Sample	mRNA	Quantification fator in the abscess	Quantification fator in the culture	Normalized Quantification fator in the abscess × 1000	Normalized Quantification fator in the culture × 1000	Expression multiplicity
Animal 1	*rna16S*	12,213	1,693,182			
	*nanH*	10,245	516,504	838,860	305,049	2750
	*Cpp*	7786	449,959	637,517	265,748	2399
	*Pld*	7203	754,682	589,781	445,718	1323
	*sodC*	12,990	710,035	1,063,621	419,349	2536
	*spaC*	*11,330*	*611,099*	*927,700*	*360,917*	*2570*
Animal 2	*rna16S*	10,751	3,587,966			
	*nanH*	14,466	843,655	1,345,549	235,135	5722
	*Cpp*	11,368	756,508	1,057,390	210,846	5015
	*Pld*	11,753	939,822	1,093,201	261,937	4174
	*sodC*	17,348	1,055,413	1,613,617	294,154	5486
	*spaC*	*14,273*	*1,025,113*	*1,327,597*	*285,709*	*4647*
Animal 3	*rna16S*	12,109	2,490,341			
	*nanH*	17,684	512,642	1,460,401	205,852	7094
	*Cpp*	13,546	429,309	1,118,672	172,390	6489
	*Pld*	15,912	460,927	1,314,064	185,086	7100
	*sodC*	20,697	577,854	1,709,225	232,038	7366
	*spaC*	*20,171*	*601,448*	*1,665,786*	*241,512*	*6897*
Animal 4	*rna16S*	24,930	4,981,860			
	*nanH*	8376	161,958	335,981	32,510	10,335
	*Cpp*	7063	129,867	283,313	26,068	10,868
	*Pld*	7327	213,745	293,903	42,905	6850
	*sodC*	9982	215,407	400,401	43,238	9260
	*spaC*	*8903*	*189,375*	*357,120*	*38,013*	*9395*
Animal 5	*rna16S*	13,377	2,012,331			
	*nanH*	18,657	187,995	1,394,707	93,422	14,929
	*Cpp*	15,847	196,526	1,184,645	97,661	12,130
	*Pld*	17,692	254,409	1,322,569	126,425	10,461
	*sodC*	22,484	260,336	1,680,795	129,370	12,992
	*spaC*	20,163	292,056	1,507,289	145,133	10,386

For relative DNA quantification (and, by extension, mRNA), a quantification value was determined for each of the six genes studied as follows: quantification factor = 10^W^, in which w = [linear coefficient − (Ct × angular coefficient)]. By dividing the values of the quantization factors corresponding to each genes by the respective values of the housekeeping gene, we obtained the relative expression values of the studied genes. Finally, the ratio of abscess values/culture was calculated to indicate the number of times a particular gene was more highly expressed in an abscess.

### Statistical analysis

The Mann–Whitney test was used to compare the expression of each gene in culture with expression in abscesses. The relative expression of each gene in each of the five animals was compared using the Kruskal–Wallis test. p values < 0.05 were considered significant.

## Results

### Quality assessments of RNA extraction and cDNA production

**Fig. 2:**
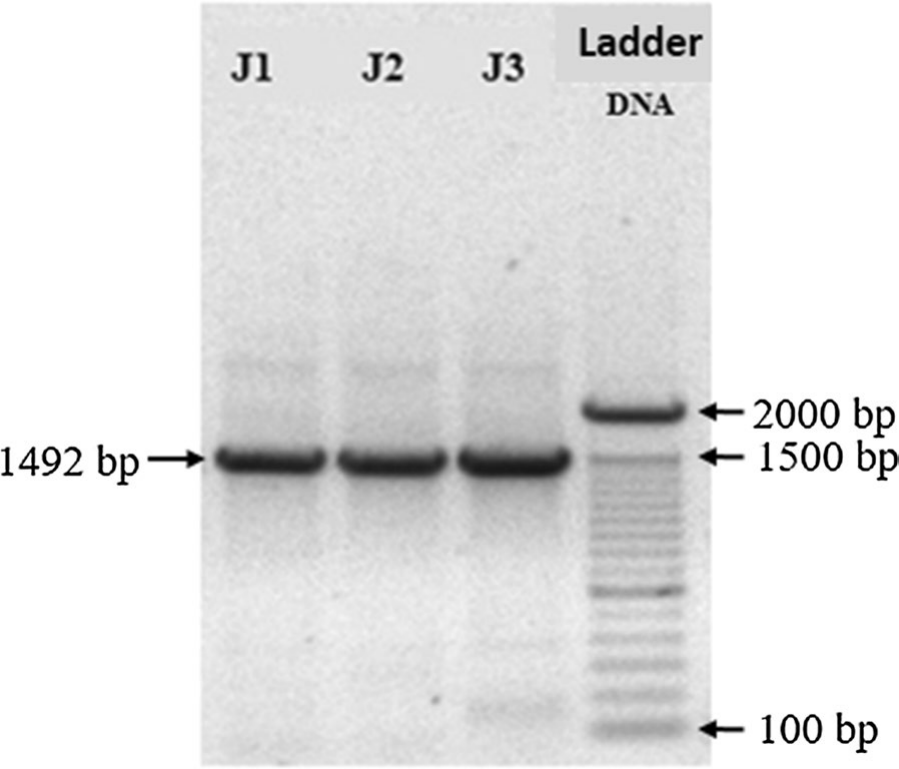
1% agarose gel used to show the 1492 bp bands found in abscess of three animals (J1, J2 and J3) naturally infected by *Corynebacterium pseudotuberculosis*

Figure 2 depicts the 1.0% agarose gel used to verify the quality of the amplicons produced by conventional PCR following RNA extraction and cDNA production from three different samples containing a virulent strain of *C. pseudotuberculosis*.

The DNA ladder varies between 100 and 2000 base pairs (bp), with greater visibility seen on 2000, 1500 and 600 bp. Three bands were seen around 1500 bp, characterizing the amplicon with 1492 bp, demonstrating the suitability of the protocols used in this study for the extraction and efficient production of cDNA by real-time PCR.

### Gene expression in vitro and in vivo

With respect to messenger RNA synthesis, all of the genes evaluated had in vitro expression. Table 1 shows the quantification of *rRNA16S*, *nanH*, *cpp*, *pld*, *sodC*, and *spaC* genes in abscesses and their respective cultures, corresponding normalization indices and expression ratios of abscesses compared to cultures. These same genes were also expressed in vivo, but with greater intensity.

Linear and angular coefficient values obtained from linear regression performed for the cDNA dilution curves of all genes are presented in Additional file 1, which also contains the linear regression line equations, and the formulas used to calculate the quantified variables shown in Table 1.

Average RNAm expression of the five genes expressed in abscesses and cultures varied significantly, as follows: *nanH* 811.50 ± 198.27 and 359.35 ± 75.45 (p = 0.009); *cpp* 856.31 ± 385.11 and 154.54 ± 94.34 (p = 0.0039); *pld* 922.70 ± 450.73 and 212.41 ± 153.10 (p = 0.016); *sodC* 1,293,53 ± 564.75 and 223.63 ± 145.58 (p = 0.016) and spaC 1,157,10 ± 525.13 and 214.26 ± 125.70 (p = 0.016) (Fig. 2).

The *nanH*, *cpp*, *pld*, *sodC* and *spaC* genes were more highly expressed in the abscesses than in the cultures by approximate factors, 8, 7, 6, 7, and 7, respectively, according to Table 1. The relative mean levels of expression of all genes in each animal were: 2.32 (animal 1), 5.01 (animal 2), 6.99 (animal 3), 9.34 (animal 4) and 12.18 (animal 5), respectively. The Kruskal–Wallis test demonstrated that these averages differed statistically, but a larger sample is necessary to clarify whether some biological property is responsible for these findings in the abscesses. No statistical differences were seen in the expression of five genes in the abscess of each animal evaluated separately. Table 1 shows the mean relative transcription values of the five genes, comparing in vivo and in vitro expression.

## Discussion

mRNA detection of the *nanH*, *cpp*, *pld*, *sodC*, and *spaC* genes in bacterial cells obtained from culture indicated that transcription of these genes occurred, in addition to the possible synthesis of the respective proteins. Although it is possible to transcribe genes without the corresponding translation (Houben et al. 2009), in the context of our research, the probability of this occurring in five genes simultaneously is extremely low. Additionally, it is known that the synthesis and secretion of phospholipase D and CP40 proteins in cultures of *C. pseudotuberculosis* occurs, since these substances are detected in culture supernatants (Walker et al. 1994; Wilson et al. 1995). In addition, the interference of phospholipase D in the attenuation of hemolysis caused by β-hemolysin from *Staphylococcus aureus* has been documented in vitro, which serves as an auxiliary test in the identification of *C. pseudotuberculosis*.

While NanH (neuraminidase H) is present on the surface of some bacteria, this enzyme can also be secreted, but this has yet to be proven in *C. pseudotuberculosis*. The molecular weight of the bacterial neuraminidases can approach 40 kDa, or be 80 kDa or more. As previously mentioned, the serum of naturally infected animals contained antibodies against several antigens present in the supernatant of *C. pseudotuberculosis* cultures.

These antibodies recognize antigens with a molecular weight between 31 and 32 kDa, corresponding to phospholipase D (Songer et al. 1990; Linder and Bernheimer 1978), 38–40 kDa, corresponding to CP40 protein (Walker et al. 1994; Wilson et al. 1995) and proteins weighing 20, 24–25, 27–28, 63 and 68 kDa, whose structures and functions have yet to be identified (Meyer 2003). Other antigens with molecular weights of 75, 85, 90, 108 and 125 kDa, initially detected in somatic extracts (Ellis et al. 1991; Braithwaite et al. 1993) were also seen among those secreted when this bacterium was cultured in synthetic media (Paule et al. 2004). Considering the molecular weight range of these unknown antigens weighing from 20 to 125 kDa, which is compatible with the molecular weights of some neuraminidases (including NanH), it is possible the antibodies present in diseased animals also recognize this enzyme.

The detection of SodC mRNA in bacterial cells cultured in vitro was expected, as all aerobic and aerotolerant bacteria constitutively produce superoxide dismutases when exposed to air or are allowed to grow in environments with molecular oxygen (Dahr et al. 2013). It is not known whether *C. pseudotuberculosis* secretes this enzyme which, as mentioned previously, is normally present within the cytoplasm, in the periplasmic space of gram-negative bacteria or on the surface of gram-positive cells. When secreted, the molecular weight of SodC expressed by *C. pseudotuberculosis* ranges between 20 and 125 kDa and is recognized by antibodies from infected animals (Meyer 2003).

SpaC is a protein located at the tip of the pili of at least three species of corynebacteria (Chang et al. 2011; Zasada et al. 2012; Trost et al. 2010, 2011). In *Corynebacterium diphtheriae*, SrtA sortase catalyzes the synthesis of the SpaA-type pili, consisting almost exclusively of SpaA pilin, SpaC adhesin (located at its tip) and secondary pilin called SpaB. SpaA-type pili anchorage is triggered when SrtA sortase incorporates a molecule of SpaB at the base of the pili by way of lysine-mediated transpeptidation. The final binding step of heteropolymer to murein is mediated by a second sortase, SrtF (Chang et al. 2011). In *C. diphtheria,* there are nine types of genes coding for pilins, located on an island of pathogenicity (Zasada et al. 2012). SpaC adhesin has also been described in *C. ulcerans* and *Corynebacterium pseudotuberculosis* (Trost et al. 2010, 2011). The adhesion of *C. diphtheriae* to human pharyngeal epithelial cells is critically dependent on the SpaC protein (Rogers et al. 2011). Given the degree of evolutionary proximity between these species, it is plausible that these three species share similar structure and physiology with respect to the pilli containing SpaC (Dorella et al. 2006).

From an evolutionary point of view, the advantages obtained by bacterial cells that express these proteins considered virulence factors, or which collaborate in some way to microbial aggressiveness, appears paradoxical with respect to energy. In the case of *C. pseudotuberculosis*, the gene expression profile while the pathogen is in transit, i.e. outside its natural host, remains unknown. Abscesses in the superficial lymph nodes may spontaneously rupture or may be removed by surgical drainage, both situations that favor environmental contamination. Purulent secretions protected from direct sunlight may harbor viable germs for up to 60 days, and no research has been performed on this material to characterize the gene expression profile. To some extent, if virulence-related molecules are already present in the bacterium while in transit to a host, it could be argued that rapid microbial interference could be facilitated with respect to host innate immunity. Clearly, this does not apply in all situations, since the pathogen may be present in a variety of infectious materials, which may highly differ as to the presence of organic material, dust, degree of hydration, contaminating microbiota, acidity, aeration, among others.

The mRNA expression of genes *nanH, pld, sodC,* and *spaC* detected in goat abscesses was expected, since the neuraminidases, phospholipases, superoxide dismutase and adhesins (SodC) are important bacterial virulence factors. The in vivo expression of CP40 serine protease was also expected, considering that antibodies from infected animals recognize this antigen under Western blotting (Walker et al. 1994; Wilson et al. 1995).

Neuraminidase H mRNA showed the highest relative increase in this study, followed by SodC, CP40, SpaC and phospholipase D mRNAs. It is not possible to definitively define the clinical origin of these differences in gene expression. It is worth noting that all animals selected for sample collection were adults in good physical condition, but the clinical evolution of each animal would be necessary to consider in order to more clearly define clinical origin.

The absence of variation in intra-animal mRNA expression of these genes was unexpected, since there are differences in the involvement of the synthesized molecules with respect to virulence and pathogenesis. The participation of gene transcription regulators may in some way justify this finding. In silico analysis of *C. pseudotuberculosis* FRC41 revealed the existence of 31 potential regulators of gene transcription (Trost et al. 2010) in this bacterium. Although the strain isolated from goats used in this study is not the that the cited study, it is known that there is great genetic similarity among the different samples sequenced to date.

Accordingly, the sodC gene is part of a regulon that also contains the sodA, ahpC and ahpD genes, which are all controlled by the OxyR transcription factor. However, spaC is not controlled by OxyR, but rather by GlxR, another transcription controller which also influences the transcription of genes nanH and cpp (Trost et al. 2010; Pacheco et al. 2011; Suvorova et al. 2015; Santana-jorge et al. 2016).

In sum, the expression of three of the five genes investigated herein could be activated in vivo, probably by way of the same transcription controller, GlxR (Suvorova et al. 2015). Under oxidative stress in animal tissue, the natural expression of the sodC and pld genes appears to be constitutively activated (Hodgson et al. 1999). These considerations may aid in understanding the simultaneous expression of these genes in vivo.

The *pld, cpp, nanH, sodC* and *spaC* genes of *Corynebacterium pseudotuberculosis* were found to transcribed in liquid cultures and in the caseous abscesses of goats, appearing to be constitutively expressed. The transcription of these genes within the abscesses was 6–8 times higher than in culture. No significant differences were detected with respect to the expression of the five genes studied in each animal studied, yet the means of relative gene expression among the tested animals were found to differ significantly. These data require additional confirmation in a larger number of samples, since the sample size herein was limited, thus there was a genetic expression of the bacterium in vitro, but in vivo was better observed so the virulence factors are shown to be important in the pathogenesis of the disease. It is probable that the SodC, NanH and SpaC proteins are also constitutively synthesized and, as the latter two are superficial, it may be of interest to further investigate their applicability as antigenic reinforcement in vaccines against caseous lymphadenitis.

## Additional file

**Additional file 1.** Linear, angular coefficients, regression equations and formulas for calculating the quantification factors in Table 1.
