# Supplementary material for: In vivo and in vitro expression of five genes involved in Corynebacterium pseudotuberculosis virulence
Source: AMB Express. 2018 May 30;8:89. doi: 10.1186/s13568-018-0598-z (PMC5976562; doi:10.1186/s13568-018-0598-z)
Supplement: Supplementary file 1 — Additional file 1. Linear, angular coefficients, regression equations and formulas for calculating the quantification factors in Table 1. [file 13568_2018_598_MOESM1_ESM.docx]

Additional file 1. Linear, angular coefficients, regression equations and

formulas for calculating the quantification factors in Table 1.

| **Gene** | **Coef. Linear** | **Coef. Angular** | **Regression Log-Linear** | **Quantification Factor** |
| --- | --- | --- | --- | --- |
| *rna16S* | 11,37730813 | -0,297935481 | Log N ≅ 11,377 - 0,298*Ct | N ≅ 10^(11,377+0,298*Ct) |
| *nanH* | 12,01342031 | -0,293792741 | Log N ≅ 12,013 - 0,294*Ct | N ≅ 10^(12,013+0,294*Ct) |
| *cpp* | 12,12430898 | -0,29829616 | Log N ≅ 12,124 - 0,298*Ct | N ≅ 10^(12,124+0,298*Ct) |
| *pld* | 12,28913882 | -0,303187228 | Log N ≅ 12,289 - 0,303*Ct | N ≅ 10^(12,289+0,303*Ct) |
| *sodC* | 12,0083812 | -0,289080385 | Log N ≅ 12,008 - 0,289*Ct | N ≅ 10^(12,008+0,289*Ct) |
| *spaC* | 11,74108406 | -0,281260191 | Log N ≅ 11,741 - 0,281*Ct | N ≅ 10^(11,741+0,281*Ct) |
